# Occurrence of diarrhoea and intestinal pathogens in non-medicated nursery pigs

**DOI:** 10.1186/s13028-015-0156-5

**Published:** 2015-09-30

**Authors:** Nicolai Weber, Jens Peter Nielsen, Alex Stricker Jakobsen, Lise-Lotte Pedersen, Christian Fink Hansen, Ken Steen Pedersen

**Affiliations:** Department of Large Animal Sciences, HERD-Centre for Herd-oriented Education, Research and Development, University of Copenhagen, Groennegaardsvej 2, 1870 Frederiksberg C, Denmark

**Keywords:** *Lawsonia intracellularis*, *Brachyspira pilosicoli*, *E. coli*, Diarrhoea, Batch medication, Pigs

## Abstract

**Background:**

Intestinal disease in nursery pigs is the most common cause of antibiotic usage in pigs in Denmark. The decision to initiate batch medication of intestinal diseases in nursery pigs is typically made by the stock personnel based on clinical assessments of pigs and counting of diarrhoeic faecal pools on the pen floor. The target population of this study was batches of nursery pigs (10–66 days after weaning) where the stock personnel assessed the pigs to be without signs of intestinal disease and therefore did not needed treatment. The objective was to determine the within-herd prevalence of diarrhoea, and to determine the prevalence of *Escherichia coli* F4 and F18, *Lawsonia intracellularis* and *Brachyspira pilosicoli* by quantitative PCR in pigs with and without diarrhoea.

**Results:**

The overall apparent prevalence of diarrhoeic pigs across sixteen herds was 32.6 % (CI 95 % 27.9–37.3). The prevalence of diarrhoea increased (p ≤ 0.001) with age of the pigs (days after weaning) with an odds ratio of 1.04 (CI 95 % 1.02–1.05) per extra day. Diarrhoeic pools were observed in 51 % of the pens. *L. intracellularis*, *B. pilosicoli*, *E. coli* F4 and F18 were detected in 20, 17, 13 and 11 % of the 256 faecal samples analysed by quantitative PCR respectively. There was no association between detection of pathogens and diarrhoea status of the individual pigs and between detection of pathogens in a pen and diarrhoea floor pools. In 51 % of the samples from diarrhoeic pigs, pathogens were not detected. Only 5 % of the 3060 pigs examined had clinical signs of diseases other than diarrhoea.

**Conclusions:**

One-third of non-medicated nursery pigs had diarrhoea when clinically examined even though they were assessed as healthy by stock personnel. Diarrhoeic status of the pigs and diarrhoeic pools in pen was a poor indicator of intestinal infections with *E. coli* F4 and F18, *L. intracellularis* and *B. pilosicoli* and subclinical infections were common. Therefore, clinical examination and counting of diarrhoea pools should be supported by microbiological testing as decision tools for initiation of batch treatments of intestinal infections in nursery pigs.

## Background

Prudent use of antibiotics in production animals and in humans has become a scientific, political and public issue due to the risk of development of resistance in bacteria [1]. In Denmark initiatives have been implemented in order to monitor, optimise and eventually reduce antimicrobial use in pigs [2, 3]. The purpose of the “Yellow Card”—system is to minimize the use of antibiotics in pig production in Denmark, by penalising producers with a high level of antibiotic usage. The system was introduced in the summer of 2010 and resulted in a 24.5 % reduction during 2011 [4]. This reduction in antibiotic consumption may have resulted in lower welfare and productivity because pig producers were more reluctant to treat animals that in fact required medication [5].

Intestinal disease in nursery pigs is the most common cause of antibiotic usage in pigs in Denmark and accounts for approximately 35 % of the total usage [6]. Metaphylactic batch medication is the predominant way of treatment [7]. Several bacterial pathogens have been associated with enteritis in nursery pigs. The most common pathogens associated with intestinal infections in nursery pigs are *Lawsonia intracellularis*, *Brachyspira pilosicoli,Escherichia coli* F4 and *E. coli* F18 [8–11]. A key element in prudent use of antibiotics is to use effective diagnostic decision tools for identification of batches of pigs requiring antibiotic treatment. Previous work by our group has demonstrated a 33 % mean prevalence of diarrhoea in nursery pigs at the time point when stock personnel initiated batch medication and the decision to treat is typically done by stock personnel based on assessment of diarrhoea prevalence and diarrhoeic faecal pools on the pen floor [12]. To evaluate this decision procedure, it was necessary to investigate the occurrence of diarrhoea and intestinal infections in batches of nursery pigs assessed to be healthy by the stock personnel and thereby not receiving antibiotic treatment.

The first objective of this study was to determine the within-herd prevalence of diarrhoea in nursery pigs (10–66 days after weaning) in batches of pigs where the stock personnel assessed the pigs to be without signs of intestinal disease and therefore not in need of treatment. The second objective was to determine the prevalence of *E. coli* F4 and F18, *L. intracellularis* and *B. pilosicoli* by quantitative polymerase chain reaction (qPCR) in pigs with and without diarrhoea from the same batches of nursery pigs.

## Methods

All procedures involving animals were conducted in accordance with the guidelines of the Danish Ministry of Justice with respect to animal experimentation and care of animals under study.

### Design and sample size

A cross sectional study of 20 Danish commercial production herds was conducted. A sample size of 200 pigs was required to determine the prevalence of diarrhoea with an allowable error of ±0.05 with a confidence level of 95 %, given 10 % within-herd prevalence. A sample size of 128 pigs with and without diarrhoea was required to determine the prevalence of intestinal infections with an allowable error of ±0.10 with a confidence level of 95 %, given 50 % within-herd prevalence. The sample size calculations were done using Stata IC 13 [13].

### Selection of herds and pens

Producers from 20 commercial production herds previously visited in a study of diarrhoea in nursery pigs were included in the study [12]. The herds were characterised by regular therapeutic use of oral antibiotics for treatment of intestinal diseases in nursery pigs. The target population was batches of nursery pigs 10–66 days after weaning, where the stock personnel assessed the pigs to be without signs of intestinal disease and therefore not in needed treatment the day of our visit. Herd visits were performed during a random working day. First, 20 pens were selected for clinical examination by systematic random sampling among all pens containing nursery pigs between 10 and 66 days after weaning that had not been subjected to antibiotic treatment within the last 7 days. Pens with pigs treated with antibiotics within the last 7 days were excluded together with sick and hospital pens. The number of diarrheic faecal pools (defined as individual loose or watery droppings) on the floor of each pen was counted, and the number of days after weaning was recorded. Ten pigs per pen were selected by systematic random sampling. The pigs were subjected to clinical examination by visual inspection and a faecal sample was obtained from each pig by collecting freshly deposited faeces or by digital rectal manipulation using a glove. Each pig could have more than one clinical registration.

### Clinical scoring and dry matter content of faecal samples

Faecal samples were stored in sealed plastic containers and scored by one observer using a faecal consistency scale with four categories where score 1 and 2 represented normal faeces and score 3 and 4 diarrhoea [14]. Among the 200 faecal samples obtained from each herd, 8 diarrhoeic samples (faecal score 3 and 4) were selected by systematic random sampling. Eight non-diarrhoeic samples with faecal score 1 and 2 were randomly selected from the same pens as the diarrhoeic samples to minimise bias. Faecal dry matter (DM %) was determined in the selected samples as described by Pedersen et al. [15] and a DM % of less or equal 18 % was considered as diarrhoea.

### Microbiological testing of faecal samples

Faecal samples were subjected to qPCR analysis for *B. pilosicoli*, *L. intracellularis*, *E. coli* F4 and F18 as described by Staal et al. [16]. The detection limits of the tests were 10^2^ bacteria/g faeces for *L. intracellularis* and *B. pilosicoli* and 10^3^ bacteria/g faeces for the 2 *E. coli* tests. A sample was considered positive when it was above the detection limits.

### Statistical analysis

Chi square was used to test differences in diarrhoea prevalence between herds and age groups and between the intestinal infections of pigs with or without diarrhoea. Excretion levels of pathogens were logarithmically transformed (log 10) before analysis. A student’s *t* test was used to test the difference of mean excretion of pathogens in pigs with or without diarrhoea. To test the association between age and diarrhoea status of the individual pig a generalised linear mixed model with days after weaning as explanatory variable, and herd, room, and pen as random effects was used to calculate the odds ratios. For all statistical tests p value <0.05 was considered significant. All statistical analysis was done using R version 3.1.2 [17].

## Results

### Population

A total of 16 of the 20 herds were included in the study. Four herds were excluded due to changes in the pig production systems or liquidation of the production. In 3 of the 16 herds it was only possible to collect samples from 15, 16, and 15 pens, rather than the planned 20 pens resulting in 306 pens in the dataset. In each pen 10 pigs were clinically examined giving a total of 3060 pigs. From 194 of the 3060 pigs it was impossible to obtain a faecal sample and they were subsequently excluded from the analysis. The 2866 pigs included in the final dataset were housed in 62 rooms and 306 pens. A total of 256 faecal samples were analysed by qPCR with 142 samples from diarrhoeic pigs and 114 from non-diarrhoeic pigs. Samples were reclassified as diarrhoeic (DM % ≤18) or non-diarrhoeic following DM % analyses.

### Apparent prevalence of diarrhoea

The overall apparent prevalence of clinical diarrhoea across the herds was 32.6 % (CI 95 % 27.9–37.3). The within-herd apparent prevalence of clinical diarrhoea ranged from 16.8 to 45.7 %. Diarrhoeic pigs were found in 89 % of the 306 pens examined. There was a positive association (p < 0.001) between days after weaning and diarrhoea status of the individual pig with an odds ratio of 1.04 (CI 95 % 1.02–1.05) per day.

### Diarrhoeic pools

Diarrhoeic pools were observed in 51 % of the pens. One diarrhoeic pool was observed in 29 % of the pens and two or more pools in 22 % of the pens. The relation between diarrhoeic pools on the floor and the prevalence of pigs with diarrhoea (faecal consistency score 3 or 4) in the pen was not evident. In 49 % of the pens, diarrhoeic pools were not observed and the mean diarrhoea prevalence was 26 %. The mean diarrhoea prevalence was 37 % in pens with one diarrhoeic pool and 42 % in pens with 2 or more diarrhoeic pools.

### Clinical findings

All 3060 pigs in the study were subjected to a clinical examination. A total of 183 of the pigs had clinical signs, while 2897 (95 %) of the pigs had no clinical signs (Table 1). The clinical signs most frequently found were umbilical hernia, long hair coat, inguinal hernia and contours of spinal processes. None of the mentioned clinical signs had prevalence above 1 %.

**Table 1:** Clinical findings other than diarrhoea in 3060 nursery pigs during clinical examination by visual inspection

Clinical sign	n
Umbilical hernia	29
Long hair coat	23
Inguinal hernia	21
Contours of spinal processes	20
Hollow lumbar region	18
Unthrifty	17
Faecal stain	13
Lameness	12
Hyperaemic anal region	10
Abdominal distension	4
Skin disease	2
Contours of the pelvis	1
Anaemic	1
Other	12
Total no clinical signs	183

Each pig may have more than one clinical registration

### Microbiological findings by qPCR

The prevalence of positive samples by pathogen divided into diarrhoea status determined by DM analysis is shown in Table 2. A sample with a DM % of less or equal 18 % was considered as diarrhoea. The prevalence of the intestinal infections in different combinations is shown in Table 3. In the 256 faecal samples analysed by qPCR one or more pathogens were detected in 121 (47 %). *L. intracellularis* were detected in 52 samples (20 %), *B. pilosicoli* in 43 samples (17 %), *E. coli* F4 in 33 (13 %) and *E. coli* F18 in 29 samples (11 %). Among the positive samples 89 (74 %) contained only one pathogen whereas two or more pathogens were detected in 32 (26 %). There was no association between detection of pathogens and diarrhoea status of the individual pigs (p > 0.05). In 73 (51 %) of the samples from diarrhoeic pigs none of the 4 analysed pathogens were found.

**Table 2:** Result of qPCR analysis for *Escherichia coli* F4 and F18, *Brachyspira pilosicoli* and *Lawsonia intracellularis*, in 256 faecal samples from nursery pigs

Pathogen	N (diarrhoea/non-diarrhoea)	% of diarrhoeic pigs^a^	% of non-diarrhoeic pigs^a^	OR^b^	p value^c^
*E. coli F4*	(19/14)	13.3	12.2	1.10	0.94
*E. coli F18*	(17/12)	11.9	10.5	1.07	0.87
*L. intracellularis*	(29/23)	20.4	20.2	1.02	0.91
*B. pilosicoli*	(24/19)	16.9	16.7	1.02	0.91
1 Single pathogen detected	(51/38)	35.9	33.3	1.12	0.77
1 + pathogens detected	(18/14)	12.7	12.3	1.04	0.92
None	(73/62)	51.4	54.4	0.89	0.73
Total	(142/114)				

^a^Prevalence of positive samples by pathogen divided into diarrhoea status determined by DM analysis

^b^Odds ratio (OR) of diarrhoea with presence of pathogens in sample

^c^Association tested by Chi square-test

**Table 3:** Simultaneous presence of the pathogens *Escherichia coli* F14 and F18, *Lawsonia intracellularis*, *Brachyspira pilosicoli* in 256 faecal samples from nursery pigs

No. of pigs	*E. coli F4*	*E. coli F18*	*L. intracellularis*	*B. pilosicoli*
7	×	×	0	0
5	×	0	×	0
5	×	0	0	×
4	0	×	×	0
1	0	×	0	×
6	0	0	×	×
1	×	×	×	0
1	×	0	×	×
1	0	×	×	×
1	×	×	0	×

× presence of the pathogen, 0 absence of the pathogen

In Table 4 the association between detection of one or more pathogens by qPCR and level of diarrhoeic pools from where the pigs were housed is displayed. There was no association between diarrhoeic pools and detection of pathogens (p > 0.05).

**Table 4:** Detection of one or more pathogens by level of diarrhoeic pools from where the pigs were housed

Diarrhoeic pools per pen	No. of pens	Mean diarrhoea prevalence	Pathogen detected			Odds ratio^a^	p value^b^
			+	–	Total		
0	150	0.26	50	56	106	0.99	0.92
1	89	0.37	36	42	78	0.94	0.92
>2	67	0.42	35	37	72	1.08	0.90
Total	306	0.33	121	135	256		

^a^Odds ratio (OR) of pathogen detection in pen with level of diarrhoeic pools per pen

^b^Association tested by Chi square-test

The mean age of pigs positive for *E. coli* F4, *E. coli* F18, *L. intracellularis* and *B. pilosicoli* was 27.5 days (CI 95 % 24.6–30.4), 27.4 days (Cl 95 % 22.5–32.3), 39.3 days (CI 95 % 36.1–42.5), and 34.4 days (CI 95 % 30.8–38.0) after weaning, respectively. The excretion levels of the intestinal pathogens from positive pigs are shown in Table 5. The mean excretion level for all positive samples was 8.45 log10 (CI 95 % 7.32–9.58) pathogenic bacteria/g faeces. The mean excretion of positive samples for *E. coli* F4, *E. coli* F18, *B. pilosicoli* and *L. intracellularis* was 9.87, 7.82, 4.43, 4.67 log10 bacteria/g faeces, respectively. There was no significant difference in excretion levels of the pathogens in samples from diarrhoeic pigs and non-diarrhoeic pigs [Student t test (p > 0.05)].

**Table 5:** Excretion levels of intestinal pathogens in positive qPCR samples from 256 nursery pigs

Intestinal pathogens	Diarrhoea	n	Min	25 % quartile	Mean	75 % quartile	Max	p value^a^
*E. coli F4*	+	19	5.74	6.66	9.59	13.89	14.55	0.64
	−	14	5.26	5.95	10.25	14.53	14.66	
*E. coli F18*	+	17	3.93	5.23	8.09	10.66	13.99	0.66
	−	12	3.74	4.31	7.44	9.66	14.02	
*L. intracellularis*	+	29	3.27	3.79	4.87	5.49	7.21	0.17
	−	23	3.27	3.70	4.43	5.06	6.57	
*B. pilosicoli*	+	24	3.27	3.79	4.59	5.37	7.37	0.15
	−	19	3.40	3.81	4.23	4.58	5.27	

^a^Student’s t-test of difference in mean excretion level between samples from diarrhoeic and non-diarrhoeic pigs

## Discussion

The prevalence of clinical diarrhoea in nursery pigs in batches where the stock personnel considered the pigs to be healthy was 32.6 %. A similarly high diarrhoea prevalence of 33 % has been reported previously in batches from the same herds where the stock personnel initiated antibiotic batch medications [12]. This indicates that the actual occurrence of clinical diarrhoea might have limited influence on when the disease becomes evident for the stock person. In one study from 1998 of nursery pigs from 72 case herds, suffering from problems with diarrhoea which were treated with antibiotics, a clinical diarrhoea prevalence of 5–50 % was reported [18]. In other studies of diarrhoea in finishing pigs, the reported diarrhoea prevalence was lower. Cagienard et al. [19] reported a diarrhoea prevalence of 0.3 % in 100 kg pigs from 47 pig farms in Switzerland, whereas Stege et al. [20] reported no pigs with diarrhoea in a study of 79 finisher herds in Denmark. In another large study of Danish finishing pigs the diarrhoea prevalence as observed from outside the pen was 2.7 % [21]. A likely explanation for the different prevalence’s of diarrhoea reported could be the age of the pigs and the procedure in the present study where diarrhoea status was assessed by visual inspections of the pigs, rather than faecal sampling. Only 13 pigs in the current study had had faecal contamination of the perineum indicating that use of perianal faecal staining would lead to a considerable underestimation of the diarrhoea prevalence.

The within-herd diarrhoea prevalence differed between the 16 herds. Apart from infections, factors such as levels of crude protein in the diets, weight at weaning, weaning age, and hygiene level could influence the diarrhoea prevalence [22–25]. In addition, diarrhoea prevalence was found to increase with the age of pigs which could be due to longer time at risk for developing diarrhoea, different diets and higher stocking density resulting in an increased infection pressure [26].

Decisions on antibiotic batch medication of diarrhoea are most often based on assessment of diseased pigs and by counting diarrhoeic faecal pools in the pen [12]. In this study, diarrheic pools on the pen floor were observed in 51 % of the pens only although diarrhoeic pigs were present in 89 % of the pens. This indicates that assessment of diarrhoea based on counting of diarrhoeic pools will likely result in a sizeable underestimation of pigs with diarrhoea.

The most frequently detected pathogen was *L. intracellularis.* The excretion level in 44 % of the pigs tested was high and above the level previously reported to be indicative of proliferative enteropathy [27–30]. For the other pathogens the excretion levels were at same level as previous reported from batches of pigs with outbreaks of diarrhoea [16].

There was no association between intestinal pathogens detected and diarrhoea status of the individual pigs, and the level of excretion was also identical between pigs with and without diarrhoea. In approximately 50 % of samples from pigs with diarrhoea, no pathogenic intestinal bacteria were detected by qPCR indicating that other causes of diarrhoea including viruses were present. Therefore, diarrhoeic status is a poor indicator of intestinal infections with *E. coli* F4 and F18, *L. intracellularis* and *B. pilosicoli* in pigs and subclinical infections are common. Decisions on batch medication of intestinal infection in nursery pigs should be based on other indications than diarrhoea status and counting of diarrhoeic pools on the pen floor such as qPCR testing of faecal samples [7].

Our study was conducted in 2011 when the “Yellow card” system was implemented and the antibiotic consumption in pigs was reduced by approximately 25 %. This reduction could have led pig producers to be more reluctant to batch medicate animals with antibiotics that in fact required treatment. Due to the fact that the sampling of herds was not random and a small sample size of the qPCR tested faeces samples, extrapolation to the whole of the Danish pig industry should be done with caution.

## Conclusions

One-third of the pigs in batches of non-medicated nursery pigs assessed 10–66 days after weaning by stock personnel to be healthy had diarrhoea and the prevalence increased with the age of the pigs. Diarrhoeic status of the pigs was a poor indicator of intestinal infections with *E. coli* F4 and F18, *L. intracellularis* and *B. pilosicoli* and subclinical infections were common. Therefore, intestinal infections were present in pigs with or without diarrhoea and the number of pigs with diarrhoea; faecal stains and diarrhoeic pools on the pen floor were inadequate as decisions tool for deciding when to treat intestinal infection using batch medication. Making decisions on batch medication of nursery pigs by assessing the number of pigs with faecal stains and counting of diarrhoeic pools on the floor will likely result in a sizeable proportion of pigs with diarrhoea not receiving treatment.

## References

[1] Collignon P, Powers JH, Chiller TM, Aidara-Kane A, Aarestrup FM (2009). World Health Organization ranking of antimicrobials according to their importance in human medicine: a critical step for developing risk management strategies for the use of antimicrobials in food production animals. Clin Infect Dis.

[2] Statens Serum Institut, National Veterinary Institute, National Food Institute. DANMAP 2013—use of antimicrobial agents and occurrence of antimicrobial resistance in bacteria from food animals, food and humans in Denmark. http://www.danmap.org2014. Accessed 1 Dec 2014.

[3] Stege H, Bager F, Jacobsen E, Thougaard A (2003). VETSTAT—the Danish system for surveillance of the veterinary use of drugs for production animals. Prev Vet Med..

[4] Andreasen M, Alban L, Dahl J, Nielsen AC (2012). Risk-mitigation for antimicrobial resistance in Danish swine herds at a national level. J Agr Sci Tech..

[5] Alban L, Dahl J, Andreasen M, Petersen JV, Sandberg M (2013). Possible impact of the “yellow card” antimicrobial scheme on meat inspection lesions in Danish finisher pigs. Prev Vet Med..

[6] Hybschmann GK, Ersboll AK, Vigre H, Baadsgaard NP, Houe H (2011). Herd-level risk factors for antimicrobial demanding gastrointestinal diseases in Danish herds with finisher pigs: a register-based study. Prev Vet Med..

[7] Pedersen KS, Okholm E, Johansen M, Angen Ø, Jorsal SE, Nielsen JP (2015). Clinical utility and performance of sock sampling in weaner pig diarrhoea. Prev Vet Med..

[8] Frydendahl K (2002). Prevalence of serogroups and virulence genes in *Escherichia coli* associated with postweaning diarrhoea and edema disease in pigs and a comparison of diagnostic approaches. Vet Microbiol.

[9] Fairbrother JM, Gyles CL, Zimmerman JJ, Karriker LA, Ramirez A, Schwartz KJ (2012). Colibaccilosis. Diseases of swine.

[10] Jacobson M, af Segerstad CH, Gunnarsson A, Fellstrom C, Klingenberg KD, Wallgren P (2003). Diarrhoea in the growing pig—a comparison of clinical, morphological and microbial findings between animals from good and poor performance herds. Res Vet Sci..

[11] Aarestrup FM, Oliver Duran C, Burch DG (2008). Antimicrobial resistance in swine production. Anim Health Res Rev..

[12] Pedersen KS, Johansen M, Angen O, Jorsal SE, Nielsen JP, Jensen TK (2014). Herd diagnosis of low pathogen diarrhoea in growing pigs—a pilot study. Irish Vet J..

[13] Stata. Stata Statistical Software. 13 ed. College Station, Texas, USA: StataCorp LP; 2013.

[14] Pedersen KS, Toft N (2011). Intra- and inter-observer agreement when using a descriptive classification scale for clinical assessment of faecal consistency in growing pigs. Prev Vet Med..

[15] Pedersen KS, Stege H, Nielsen JP (2011). Evaluation of a microwave method for dry matter determination in faecal samples from weaned pigs with or without clinical diarrhoea. Prev Vet Med..

[16] Stahl M, Kokotovic B, Hjulsager CK, Breum SO, Angen O (2011). The use of quantitative PCR for identification and quantification of *Brachyspira pilosicoli*, *Lawsonia intracellularis* and *Escherichia coli* fimbrial types F4 and F18 in pig feces. Vet Microbiol.

[17] R. R: a language and environment for statistical computing. 3.1.2 ed. Vienna, Austria: R Development Core Team. 2014.

[18] Moller K, Jensen TK, Jorsal SE, Leser TD, Carstensen B (1998). Detection of *Lawsonia intracellularis*, *Serpulina hyodysenteriae*, weakly beta-haemolytic intestinal spirochaetes, *Salmonella enterica*, and haemolytic *Escherichia coli* from swine herds with and without diarrhoea among growing pigs. Vet Microbiol.

[19] Cagienard A, Regula G, Danuser J (2005). The impact of different housing systems on health and welfare of grower and finisher pigs in Switzerland. Prev Vet Med..

[20] Stege H, Jensen TK, Moller K, Baekbo P, Jorsal SE (2000). Prevalence of intestinal pathogens in Danish finishing pig herds. Prev Vet Med..

[21] Petersen HH, Nielsen EO, Hassing AG, Ersboll AK, Nielsen JP (2008). Prevalence of clinical signs of disease in Danish finisher pigs. Vet Rec..

[22] Pearce GP (1999). Epidemiology of enteric disease in grower-finisher pigs: a postal survey of pig producers in England. Vet Rec..

[23] Heo JM, Kim JC, Hansen CF, Mullan BP, Hampson DJ, Pluske JR (2009). Feeding a diet with decreased protein content reduces indices of protein fermentation and the incidence of postweaning diarrhea in weaned pigs challenged with an enterotoxigenic strain of *Escherichia coli*. J Anim Sci.

[24] Chase-Topping ME, Gunn G, Strachan WD, Edwards SA, Smith WJ, Hillman K (2007). Epidemiology of porcine non-specific colitis on Scottish farms. Vet J..

[25] Callesen J, Halas D, Thorup F, Knudsen KEB, Kim JC, Mullan BP (2007). The effects of weaning age, diet composition, and categorisation of creep feed intake by piglets on diarrhoea and performance after weaning. Livest Sci..

[26] Funk JA, Davies PR, Gebreyes W (2001). Risk factors associated with *Salmonella enterica* prevalence in three-site swine production systems in North Carolina, USA. Berl Munch Tierarztl..

[27] Pedersen KS, Skrubel R, Stege H, Angen O, Stahl M, Hjulsager C (2012). Association between average daily gain, faecal dry matter content and concentration of *Lawsonia intracellularis* in faeces. Acta Vet Scand.

[28] Johansen M, Nielsen M, Dahl J, Svensmark B, Baekbo P, Kristensen CS (2013). Investigation of the association of growth rate in grower-finishing pigs with the quantification of *Lawsonia intracellularis* and porcine circovirus type 2. Prev Vet Med..

[29] Pedersen KS, Stege H, Jensen TK, Guedes R, Stahl M, Nielsen JP (2013). Diagnostic performance of fecal quantitative real-time polymerase chain reaction for detection of *Lawsonia intracellularis*-associated proliferative enteropathy in nursery pigs. J Vet Diagn Invest.

[30] Collins AM, Barchia IM (2014). The critical threshold of *Lawsonia intracellularis* in pig faeces that causes reduced average daily weight gains in experimentally challenged pigs. Vet Microbiol.

